# Predicting self-harm in an ethnically diverse sample of lesbian, gay and bisexual people in the United Kingdom

**DOI:** 10.1177/0020764020908889

**Published:** 2020-03-03

**Authors:** Zaqia Rehman, Barbara Lopes, Rusi Jaspal

**Affiliations:** 1 Faculty of Health & Life Sciences, De Montfort University, Leicester, UK; 2 CINEICC, Faculdade de Psicologia e de Ciências da Educação, Universidade de Coimbra, Coimbra, Portugal; 3 School of Social Sciences, Nottingham Trent University, Nottingham, UK

**Keywords:** Self-harm, discrimination, victimization, internalized homophobia, lesbian, gay, bisexual

## Abstract

**Background:**

Poor mental health is prevalent in lesbian, gay and bisexual (LGB) people due in part to social stigma. The social, psychological and clinical risk factors for self-harm among LGB people are unclear, which limits our ability to predict when and how this will occur and, crucially, how to prevent it.

**Aims:**

Drawing on the cognitive-behavioral approach in clinical psychology, this study identifies the predictors of self-harm in LGB people in the United Kingdom.

**Results:**

Women, lesbians, those with lower income and younger people were more likely to engage in self-harm. Self-harmers exhibited much more discrimination, LGB victimization and, thus, internalized homophobia and depressive symptomatology than non-self-harmers. The structural equation model showed direct effects of age and gender, and indirect effects of income and sexual orientation, on self-harm, through the mediating variables of discrimination, LGB victimization and internalized homophobia.

**Conclusions:**

Consistent with the cognitive-behavioral model, the results indicate that exposure to situational stressors can increase the risk of developing a self-hatred and depressive psychological self-schema, resulting in greater risk of self-harm as a maladaptive coping strategy. An integrative clinical intervention for enhancing psychological wellbeing in LGB people is proposed to mitigate the risk of self-harm in this population.

## Introduction

In the United Kingdom, there has been significant progress in the visibility, acceptance and rights of lesbian, gay and bisexual (LGB) people. Homophobia appears to be waning. More LGB people are coming out about their sexual identities than ever before. Many seek public recognition of their same-sex unions through civil partnership and marriage. Yet, over the last 5 years, there has also been a significant increase in the number of LGB people who report hate crime because of their sexual orientation (Stonewall, 2017). Furthermore, LGB people experience significant inequalities in relation to mental health when compared to heterosexual people, including higher levels of depression, suicidal ideation, and self-harm (King et al., 2008). This higher prevalence of poor mental health has been attributed to the stigma and prejudice that many LGB people continue to experience and anticipate, in spite of the social progress made.

Self-harm is a complex clinical variable, which reflects one’s desire to inflict intentional harm on oneself without the intention to end one’s life (Hawton et al., 2002). Although the term ‘non-suicidal self-injury’ is also commonly used in research, in this study, we use the term ‘self-harm’. Although not all people who self-harm wish to end their lives, there is a strong association between self-harm and depressive symptomatology (including suicidal ideation) – especially in LGB people (see King et al., 2008). There is also an observed empirical association between self-harm and poor problem-solving ability (Slee et al., 2008). The social, psychological and clinical risk factors for self-harm among LGB people are unclear, which limits our ability to predict when and how this will occur and, crucially, how to prevent it.

Accordingly, this study draws on the cognitive-behavioral model (Beck, 1976) to examine the associations between social ‘triggers’ (e.g., discrimination), psychological self-schemata (e.g., internalized homophobia) and the clinical variable of self-harm in an ethnically diverse sample of LGB people in the United Kingdom.

### Mental health in sexual minorities

Minority stress theory (Meyer, 2003) postulates that LGB people experience situational stressors, such as discrimination and hate crime, due to their stigmatized sexual identities, which in turn can undermine mental health outcomes. In a survey of 5,375 LGB people in the United Kingdom, it was found that 21% had experienced a hate crime, 17% had faced discrimination in a café, restaurant or bar, and 10% had experienced online abuse because of their sexual orientation (Stonewall, 2017). Moreover, there is a higher prevalence of childhood adversity, such as bullying and childhood sexual abuse, among LGB people (Warbelow & Cobb, 2014). These stressors are associated with the onset of poor mental health.

LGB people face disproportionately high levels of depressive symptomatology, including depression, anxiety and psychological distress (Russell & Fish, 2016). Depressive symptomatology shows onset early on in the life course – especially during adolescence which is a period characterized by significant change and the need for adaptation. As LGB people are exposed to homophobia, they may uncritically accept it and come to endorse negative attitudes toward their sexual orientation – this psychological self-schema (which is often referred to as ‘internalized homophobia’) is characterized by self-depreciative, self-hatred cognitions (Williamson, 2000). There is evidence of an association between internalized homophobia and poor mental health outcomes (Newcomb & Mustanski, 2010).

The cognitive-behavioral model (Beck, 1976) suggests that, in response to adverse events and psychological experiences, individuals attempt to cope. There is a high prevalence of maladaptive, potentially destructive behaviors in LGB people with depressive symptomatology. These include alcohol misuse, substance misuse and self-harm – these behaviors, though clearly maladaptive in the long term, may be enacted in an attempt to cope (see King et al., 2008; Liu & Mustanski, 2012). In some cases, these coping strategies allow one to distance oneself psychologically from the threatening stimulus and, in others, they constitute a form of self-punishment which alleviates negative affect associated with the threat (e.g., guilt and shame) (Liu & Mustanski, 2012; Slee et al., 2007). This study focuses on the clinical variable of self-harm.

### Self-harm in sexual minorities

Self-harm is defined as the intentional destruction of one’s own bodily tissue without suicidal intent (Liu & Mustanski, 2012). This can include many activities, including skin-cutting, scratching, burning, and beating oneself with objects. A consistent finding across many studies is that LGB people are much more likely to engage in self-harm than heterosexuals (e.g., Björkenstam et al., 2016; King et al., 2008). One systematic review of 10 studies revealed a 39.1%–59.4% prevalence of self-harm in LGB people, compared to 12%–23% among heterosexual people (McCartney, 2016).

Self-harm appears to be a response to psychological adversity. Almeida et al. (2009) found that LGB youth scored significantly higher on depressive symptomatology and that they were more than three times more likely to report self-harm, whose risk was elevated by discrimination. A study of 246 lesbian, gay, bisexual and transgender (LGBT) youth in the United States (Liu & Mustanski, 2012) revealed that a history of LGB victimization is associated with greater self-harm. Moreover, McDermott et al. (2008) found that LGB people may feel unable to access social support and, thus, employ individualized strategies for avoiding shame associated with their sexual orientation, rendering them vulnerable to maladaptive coping behaviors, such as self-harm.

Some research suggests that the antecedent of self-harm is not discrimination per se, but rather emotional dysregulation (Fraser et al., 2018). Individuals who are less able to regulate their emotional responses to potentially stressful events and situations may resort to self-harm. In their study of self-harm among university students, Taylor et al. (2018) found that LGB orientation was associated with increased risk of self-harm and that this relationship was mediated by self-esteem. This suggests that LGB people with decreased self-esteem are at especially high risk of harming themselves.

It appears that some socio-demographic traits are more associated with self-harm than others. Younger LGB people seem to be at greater risk. In her study of 219 LGB youths, Robinson (2018) found a 53% prevalence of self-harm in a younger sample and suggested that peer connectedness was associated with increased odds of engaging in self-harm. A possible explanation is that self-harm is actually quite prevalent in younger people and that LGB young people may be experiencing connectedness with others who are engaging in self-harm and, thus, contributing to a ‘norm’ in relation to this coping behavior.

Gender is also an important variable. Research consistently shows that women are at higher risk of self-harm than men (see Hawton et al., 2002). Some studies suggest that gay men appear to be at higher risk of self-harm compared to lesbian women (e.g., Almeida et al., 2009; Garofalo et al., 1999), while studies indicate lesbian and bisexual women to be at greater risk (Bostwick et al., 2014). In their study of lesbian, bisexual and heterosexual college students in the United States, Kerr et al. (2013) found that lesbian and bisexual women reported higher levels of self-harm and higher engagement with mental health services than heterosexual women. Björkenstam et al. (2016) found that lesbian and bisexual women exhibited an earlier age of onset of intentional self-harm compared to other groups. In their study of 14,371 college students, Whitlock et al. (2011) found that lesbian and bisexual women were more likely to engage in self-harm behaviors than gay and bisexual men (see also Bostwick et al., 2014).

### The cognitive-behavioral approach

**Figure 1.**
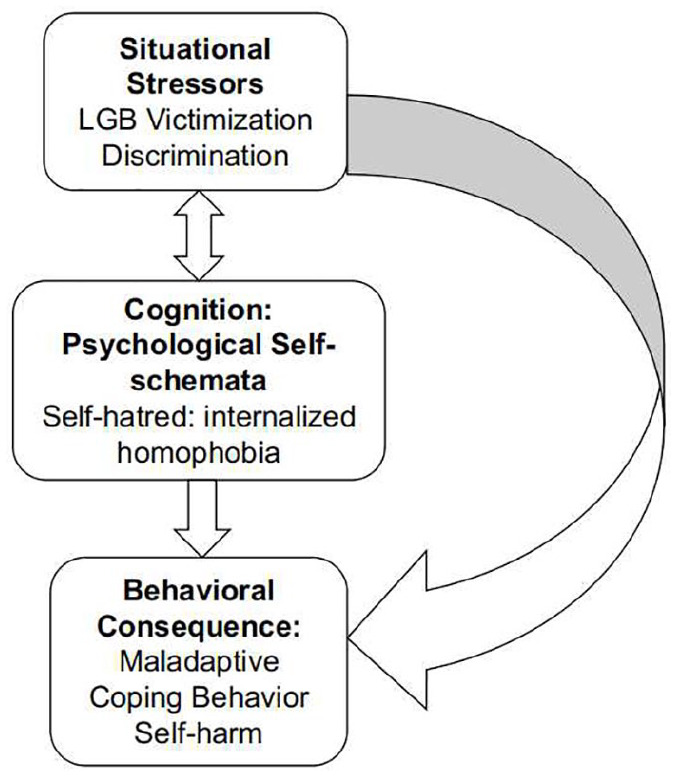
Cognitive and behavioral model of the relationships between situational stressors, internalized homophobia and self-harm in British LGB (adapted from Beck, 1976).

The cognitive-behavioral approach to psychopathology (Figure 1) provides an integrative framework within which situational stressors (e.g., discrimination), psychological self-schemata (e.g., internalized homophobia), coping strategies (e.g., self-harm) and psychopathology (e.g., psychological distress) can be collectively examined.

The cognitive-behavioral approach focuses on the role of cognitive processes, such as belief formation and interpretation, in affect and behavior. The approach postulates that most emotional problems arise from particular patterns of thinking and behavior that develop across the life course (Beck, 1976). For instance, rejection from one’s parents and homophobia can encourage negative and self-depreciating psychological schemata (e.g., internalized homophobia) that increase vulnerability to depressive symptomatology and self-harm (see Slee et al., 2007).

Exposure to situational stressors can lead the LGB individual to develop negative core beliefs about the self – when associated with their sexuality, these negative core beliefs can be considered internalized homophobia (Igartua et al., 2009). Core beliefs, whether negative or positive, guide everyday thinking and behavior – especially in response to specific stressful situations (Safren & Rogers, 2001). For instance, an individual who faces discrimination due to their sexual orientation may internalize this stigma, which in turn will lead them to refrain from coming out, and to rely instead on individualized strategies for coping devoid of social support.

The assumptions or elaborations associated with one’s core beliefs can lead to the activation of negative automatic thoughts (Safren & Rogers, 2001). These include negative causal attributions, which induce feelings of anxiety, low mood and fear (Hjemdal et al., 2013). Moreover, LGB individuals may experience difficulties in regulating negative emotions (e.g., guilt, fear and shame), which are associated with their sexual orientation (e.g., internalized homophobia; see Slee et al., 2007). Hence, LGB individuals who are unable to regulate effectively those feelings and thoughts and to manage the distress that they provoke may engage in self-harm as an escape from intolerable affect, that is, as a coping strategy (Slee et al., 2007). The escapist behavior (e.g., self-harm) may provide temporary respite from negative emotions associated with both discrimination (Suls & Fletcher, 1985) and self-hatred, and internalized homophobia.

Hence, a vicious circle is established with the negative core beliefs sustaining escapist and maladaptive behaviors (e.g., self-harm), which in turn confirm the negative and dysfunctional beliefs of unlovability and helplessness. Indeed, self-harm does not promote positive action or enable the individual to deal with the psychological and social stress that provokes depressive psychopathology. Self-harm serves only as a temporary strategy to escape and to communicate the distress that one is experiencing as a result of exposure to both internal and social threats.

### Aims and hypotheses

The aim of this study is to understand the predictors of self-harm in an ethnically diverse sample of LGB people by testing the following hypotheses:

H1. Female respondents should report more self-harm than males, and women with self-reported lesbian sexual orientation should report more self-harm than no self-harm compared to self-reported gay men.
H2. Younger LGB people and LGB people of low socio-economic background are more likely to report self-harm than older LGB people and LGB people of higher socio-economic background, respectively.
H3. Self-harmers should report significantly more depressive symptomatology, internalized homophobia, discrimination and LGB victimization than non-self-harmers.
H4. Discrimination and internalized homophobia should predict the variance of self-harm, with greater discrimination and internalized homophobia being associated with increased risk of self-harm.
H5. Being a younger female, of lower socio-economic background, and of lesbian sexual orientation will be associated with more LGB victimization and discrimination, which in turn will be associated with more internalized homophobia and, thus, self-harm.

## Method

### Ethics

This study received ethics approval from the Faculty of Health and Life Sciences Ethics Committee, De Montfort University, Leicester.

### Participants and procedure

A convenience sample of 289 individuals was recruited on various social media platforms and completed an online survey. Seventy-six (26%) participants were aged between 18 and 24 years; 120 (41.5%) between 25 and 34 years; 55 (19%) between 35 and 44 years and 38 (13%) 45+ years. One hundred and sixteen participants (41%) identified as male and 149 (51.6%) as female. The majority of participants self-identified as gay (*N* = 120, 41.%); 73 (25.3%) as lesbian; 49 (17%) as bisexual; 38 (13.1%) as Other and 7 (2.4%) as ‘same-gender loving’.

One hundred and eighty-eight participants (65%) self-identified as White, while 101 (34.9%) identified with one of the following Black, Asian and minority ethnic (BAME) groups: British Indian (*N* = 22, 7.6%); British Pakistani (*N* = 15, 5.2%); British Bangladeshi (*N* = 9, 3.1%); British Chinese (*N* = 2, 0.7%); any other British Asian background (*N* = 10, 3.5%); Black African (*N* = 8, 2.8%); Black African Caribbean (*N* = 6, 2.1%); any other Black/African/Caribbean background (*N* = 2, 0.7%); Other (*N* = 5, 1.7%) and Mixed Heritage (*N* = 10, 3.6%). One hundred and forty-one participants (48.8%) reported having a religion, while 148 participants (51.2%) reported no religion. (Christian: *N* = 39, 15.5%; Muslims, *N* = 33, 13.1%; Other, *N* = 16, 6.4%; Sikh, *N* = 5, 2%; Pagan, *N* = 4, 1.6%; Buddhist, *N* = 3, 1.2%; Hindu, *N* = 2, 0.8%; and Jewish, *N* = 1, 0.4%; Atheist, *N* = 78, 31%.

Eighty-seven participants (35.4%) had completed General Certificate of Secondary Education (GCSE)/A-level education; 95 (38.6%) had an undergraduate qualification and 64 (26%) a postgraduate qualification. Most participants (*N* = 82, 34.3%) reported an income of <£10,000; 78 (32.6%) between £10,000 and £24,999; 41 (17.2%) between £25,000 and £34,999 and 38 (16%) >£35,000. In terms of relationship status, 127 (43.9%) were single and 162 (56.1%) were married. For full information on the socio-demographic characteristics of this sample, please see Table 1.

**Table 1.** Socio-demographic information for the participant sample.

Variables	*N* (%)
Age	
18–24 years old	76 (26.3)
25–34 years old	120 (41.5)
35–44 years old	55 (19)
45+ years old	38 (13.1)
Ethnic groups	
White	188 (65.1)
BAME	101 (34.9)
Religion status	
No religion	74 (29.5)
Atheist	78 (31.1*)*
Religious	99 (39.4)
Education level groups	
A levels GCSE level and NVQ	87 (35.4)
Degree Graduate Education (e.g. BSc or BA)	95 (38.6)
Post-graduate Education (e.g. MSc, MA, PhD)	64 (26)
Income groups	
Less than £10,000	82 (34.3)
£10,000–£24,999	78 (32.6)
£25,000–£34,999	41 (17.2)
£35,000 or more	38 (15.9)
Relationship status	
Single	127 (43.9)
Married	162 (56.1)
Employment status	
Student	56 (23)
Employed	150 (61)
Self-employed	14 (5.7*)*
Unemployed	21 (8.5)
Retired	5 (2)
Gender	
Male	116 (40.1*)*
Female	149 (51.6)
Sexual orientation	
Gay	120 (41.5)
Lesbian	73 (25.3)
Bisexual	49 (17*)*
Same-gender loving	7 (2.4)
Other	38 (13.1)

BAME: Black, Asian and minority ethnic; GCSE: General Certificate of Secondary Education; NVQ: National Vocational Qualification.

### Measures

#### Discrimination

Discrimination was measured using the shortened version of the Everyday Discrimination Scale (Sternthal et al., 2011). The scale consists of five items which capture the frequency of discriminatory experiences, such as ‘being treated with less courtesy than others’. A higher score indicates more frequent discrimination. The scale manifested good internal reliability, α = .81.

#### LGB victimization

The Victimization Scale (D’Augelli et al., 2002) consists of 10 items. For this study, seven items of the scale were used to measure the frequency of the following types of victimization: verbal insults, threats of physical violence, threatened to tell others about your sexual identity, objects thrown, punched kicked, threatened with a knife, gun or another weapon, and sexual assault. The scale was adapted in relation to LGB victimization. A higher score indicates more LGB victimization. The scale manifested excellent internal reliability, α = .85.

#### Internalized homophobia

The Internalized Homophobia Scale (Meyer & Dean, 1998) was used to explore negative regard toward one’s sexual orientation and the avoidance of homosexual feelings. The scale consists of nine items, such as ‘I have tried to stop being attracted to women in general’ (when used in lesbian samples). A higher score indicates more internalized homophobia. The scale manifested good internal reliability, α = .90.

#### Psychological distress

The Kessler Psychological Distress Scale (Kessler et al., 2002) was used to yield a global measure of distress based on questions about anxiety and depressive symptoms that a person has experienced in the most recent 4-week period. The scale consists of 10 items. Examples of items are: ‘During the last 30 days, about how often did you feel tired out for no good reason?’ Participants indicate the frequency of these symptoms on a Likert-type scale. The scale manifested excellent internal reliability, α = .93.

#### Depression

The study employed the Center for Epidemiologic Studies Depression (CESD)-10 Self-Report Depression Scale (Radloff, 1977) to measure the frequency of current depressive symptoms from *Rarely* to *Most of the Time* (5–7 days). Examples of items are: ‘I felt that I could not shake off the blues even with help from my family or friends’. The scale consists of 20 items. The scale manifested excellent internal reliability, α = .87.

#### Suicidal ideation

Suicidal ideation was measured using the Suicidal Behaviors Questionnaire – Revised (Osman et al., 2001). The scale consists of four items, which tap into (1) lifetime suicidal ideation and behavior, (2) frequency of suicidal ideation over the last 12 months, (3) threats of suicide attempts and (4) likelihood of suicidal behavior. Higher scores reflect higher suicidal ideation. The authors suggest a cut-off point of 7 for non-clinical samples. The scale manifested satisfactory internal reliability, given the number of items, α = .60.

#### Self-harm

Self-harm was measured using the following two items from the Adult Psychiatric Morbidity Survey (McManus et al., 2016): ‘Have you ever deliberately harmed yourself in any way but not with the intention of killing yourself?’ and ‘Have you ever actually harmed yourself (e.g., taking pills, cutting your wrists)?’ Possible responses were ‘yes’ or ‘no’.

### Statistical analyses

SPSS and AMOS (IBM Corporate headquarters, New York, United States), version 20, were used to conduct the analyses. Since all variables of interest were normally distributed, parametric statistical analyses were performed. Chi-square tests were used to examine relationships between categorical independent variables (age, income, gender and sexual orientation groups) and the dependent variable of self-harm to test Hypotheses 1 and 2. Independent samples *t*-tests were conducted to examine differences between the self-harm versus no-self-harm groups for the variables of interest (clinical, psychological and victimization variables) to test Hypothesis 3.

Independent samples *t*-tests used bootstraps set at 1,000 samples for the 95% confidence interval (CI) for between groups’ mean differences to control for statistical power. The Cohen’s *d* values were reported for the effect sizes of the between groups’ mean differences. The Phi coefficients and the Cramer’s *V* were also reported to examine effect sizes of the chi-square analyses.

Regressions and mediation pathways require errors to be normally distributed, and testing of linearity (Normal Probability Plot), homoscedasticity (Plot of residuals versus predicted value), independence (Durbin–Watson statistic) of residuals, the presence of outliers (Cook’s distance < 1, *N* = 289) and multicollinearity (variance inflation factor (VIF) <2). All of these assumptions were tested and no problems were found – a binary logistic regression with a stepwise method and a bootstrap set at 1,000 samples to control for the 95% CI around the effects of the predictors on the dependent variable of self-harm was conducted to test Hypothesis 4.

Finally, a structural equation model with a bootstrap set at 200 samples was constructed to test a cognitive model of psychopathology including the predictors, mediators and the dependent variable of self-harm to test Hypothesis 5.

## Results

### Descriptive statistics

Table 2 includes information on the descriptive statistics for the continuous and categorical variables measured in this sample. Table 3 includes information on effect sizes and 95% CIs.

**Table 2.** Descriptive statistics for the participant sample.

Continuous variables	*M*	*SD*	Minimum	Maximum
Suicidal ideation	2.79	.70	1.73	4.24
Depression	13.26	6.69	0	30
Psychological distress	26.34	8.59	10	43
Internalized homophobia	3.96	.83	3	6.08
LGB victimization	12.12	4.90	2	28
Discrimination	8.85	5.32	0	25
Categorical DV				
*N* = 289	Yes	No		
Self-harm	*N* = 163 (57%)	*N* = 122 (43%)		

*SD*: standard deviation; LGB: lesbian, gay and bisexual; DV: dependent variable.

**Table 3.** Between-group differences (self-harm vs. no self-harm; income groups; age groups; sexual orientation groups and gender) for key variables and their respective 95% confidence intervals and effect sizes.

Variables	Self-harm *N* = 163		No self-harm *N* = 122		Independent samples *t*-tests Cohen’s *d*	95% CI
	*M*	*SD*	*M*	*SD*		
Depression	14.96	6.38	11.03	4.03	.074	–5.28, –2.07
Suicidal ideation	3.09	.61	2.38	.59	.090	–.857, –.562
Psychological distress	28.69	8.18	23.07	8.15	.069	–7.47, –3.36
Internalized homophobia	4.12	.85	3.75	.77	.045	–.598, –.180
Discrimination	10.11	5.49	7.18	4.59	.058	–4.05, –1.50
LGB victimization	12.95	5.34	11.04	4.03	.040	–2.94, –.657
	Self-harm		Chi-squared Phi/Cramer’s *V*		*P* value	
Age group 18–24 years old	34%		.310		.000	
Age group 25–34 years old	40%					
Age group 35–44 years old	20%					
Age group 45+ years old	5.5%					
Income group less than £10,000	41%		.183		.046	
Income group £10,000–£24,999	32%					
Income group £25,000–£34,999	13.2%					
Income group £35,000 and more	14%					
Females	68%		.193		.001	
Males	33%					
Sexual orientation: Gay	31%		.250		.003	
Sexual orientation: Lesbian	31%					
Sexual orientation: Bisexual	18%					

CI: confidence interval; *SD*: standard deviation; LGB: lesbian, gay and bisexual.

Most participants reported self-harm behavior (*N* = 163; 56%) versus (*N* = 122, 42.2%) no self-harm. Moreover, depression showed a *M* = 13.26 and *SD* = 6.69. One hundred and three (35.6%) individuals scored above the cut-off point of >16, which indicates risk of clinical depression. Psychological distress showed a *M* = 26.34 and *SD* = 8.59, suggesting moderate to high psychological distress in the sample, and suicidal ideation showed a *M* = 2.78 and *SD* = .69, indicating relatively frequent thoughts about suicide. A chi-square test showed no differences between White and BAME participants for risk of self-harm χ^2^ (1) = .057, *p* = .81; Phi = .014, *p* = .81. These results suggest that this sample of LGB, regardless of their ethnicity are vulnerable to both depressive symptomatology and self-harm.

### Hypothesis 1

#### Differences between females and males for self-harm

Results showed a significant effect of gender on self-harm. A chi-square test, χ^2^ (1) = 10.573; *p* = .000; Phi = .193, *p* = .001, suggested that females are more likely to self-harm than males, *N* = 110 (68%) females versus *N* = 53 (33%) males who self-harm. Since there was an effect of gender on self-harm, this variable was included in the model.

#### Differences between sexual orientation groups for self-harm

A further chi-square test showed that proportionally more lesbian women engage in self-harm compared to those who do not. In contrast to this, more gay men tend not to self-harm compared to those who do (χ^2^ (5) = 17.871, *p* = .003; Cramer’s *V* = .250, *p* = .003).

Indeed, 50 (31%) lesbian women engage in self-harm compared to 23 (19%) who do not, and 29 (18%) bisexuals self-harm, while 18 (15%) bisexuals do not. Fifty-one (31%) gay men self-harm, compared to 67 (55%) who do not. Since sexual orientation had an effect on self-harm, it was also included in the model.

These results support Hypothesis 1 and suggest that more females report self-harm compared to males, and that lesbians tend to report more self-harm compared to those who identify as gay or bisexual.

### Hypothesis 2

#### Differences between different income groups and age groups for self-harm

Chi-square tests showed that both income and age groups had statistically significant effects on self-harm χ^2^ (3) = 8.002, *p* = .046; Cramer’s *V* = .183, *p* = .046 and χ^2^ (3) = 27.334; *p* < .001; Cramer’s *V* = .310, *p* *<* .001, respectively.

LGB participants of the lower income group were likely to report self-harm than the higher income group. Indeed, 56 (41%) participants who reported an income of <£10,000 reported self-harm versus only 19 (14%) of those who reported an income of >£35,000 self-harmed.

More young LGB people report self-harm than older people – 56 (34%) 18- to 25-year olds and 65 (40%) 25- to 34-year olds report self-harm versus only 9 (6%) 45+ year olds. Since the socio-demographic variables of income and age groups did show significant effects on self-harm, they were included in the model.

These results support Hypothesis 2 and suggest that younger and lower income groups are more vulnerable to self-harm than older people and people with higher income.

### Hypothesis 3

#### Between-group differences (self-harm vs. no self-harm) for LGB victimization and discrimination, internalized homophobia and depressive symptomatology

Independent samples *t*-tests bootstrapped at 1,000 samples showed that there were statistically significant differences between self-harmers in this sample versus those who do not self-harm for LGB victimization *t* (255) = –3.102, *p* = .002 and discrimination *t* (256) = –4.367, *p* < .001; for internalized homophobia *t* (256) = –3.820, *p* < .001; and for the clinical variables of depression *t*(256) = –4.483, *p* < .001; suicidal ideation *t*(256) = –9.509, *p* < .001; and psychological distress *t*(256) = –5.188, *p* < .001.

LGB people who self-harm showed statistically significantly more depressive symptomatology (*M* = 14.96, *SD* = 6.38 for depression; *M* = 28.69, *SD* = 8.18 for psychological distress and *M* = 3.09, *SD* = .61 for suicidal ideation) than people who do not self-harm (*M* = 11.03, *SD* = 6.46 for depression; *M* = 23.07, *SD* = 8.15 for psychological distress and *M* = 2.38, *SD* = .59 for suicidal ideation, respectively).

Moreover, self-harmers also show more internalized homophobia (*M* = 4.12, *SD* = .85) and more LGB victimization and discrimination (*M* = 12.95, *SD* = 5.34 for LGB victimization and *M* = 10.11, *SD* = 5.49 for discrimination) than people who do not self-harm (*M* = 3.75, *SD* = .77 for internalized homophobia; *M* = 11.04, *SD* = 4.03 for LGB victimization and *M* = 7.18, *SD* = 4.59 for discrimination, respectively).

These results support Hypothesis 3 and suggest that self-harmers exhibit much more discrimination, LGB victimization and, thus, internalized homophobia and depressive symptomatology than non-self-harmers.

### Hypothesis 4

#### Binary logistic regression predicting self-harm

A binary logistic regression set at a bootstrap of 1,000 samples with the stepwise method of Wald for entering blocks of predictors was performed to ascertain which predictors predict the categorical dependent variable: self-harm (dummy coded as 0 = no vs. 1 = yes). All the predictors were continuous variables. The first set of predictors was constituted by discrimination and LGB victimization. The second block was constituted by the psychological variable of internalized homophobia. The model was statistically significant with χ^2^ (1) = 8.982, *p* = .003.

Out of the first set of predictors, only discrimination had predictive power for self-harm with a β = .097, Wald = 11.621, *p* = .001, whereas LGB victimization was taken out of the model as it had no predictive power. When internalized homophobia was inserted in the model, discrimination increased its predictive power with a β = .10, Wald = 13.181, *p* < .001 and internalized homophobia also added predictive power to the model with a β = .46, Wald = 8.585, *p* = .003.

Hence, in support of Hypothesis 4, self-harm was predicted only by discrimination and internalized homophobia, suggesting that experiences of discrimination and internalized homophobia are associated with increased risk of self-harm.

### Hypothesis 5

#### Structural equation model

**Figure 2.**
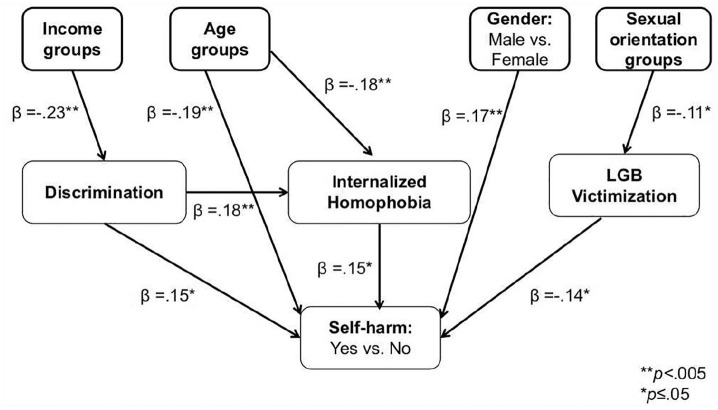
Structural equation model for the relationships between socio-demographic predictors of self-harm, and the mediators of discrimination, internalized homophobia and victimization.

Since there were significant effects of income, age, gender and sexual orientation on self-harm, these variables were inserted in the structural equation model as main predictors, followed by the following mediation variables: LGB victimization, and discrimination; and the psychological variable of internalized homophobia which represents a self-hatred psychological schema. The dependent variable was the categorical variable of self-harm. The SEM model was set with a bootstrap of 200. The model was statistically significant χ^2^ (16,289) = 193.517, *p* < .001. Model fit was good with a root mean square error of approximation (RMSEA) of .08 and a comparative fit index (CFI) > .09 (see Figure 2).

The model showed that, of the four socio-demographic predictors, only age and gender had statistically significant direct effects on self-harm (β = –.19, *p* < .001 for age and β = .17, *p* = .002 for gender, respectively). Income and sexual orientation did not have statistically significant direct effects on the variance of self-harm (β = .011, *p* = .86 and β = .068, *p* = .22, respectively). This suggested that younger females are more likely to engage in self-harm than other age and gender groups in this sample.

However, the model did show that age, income and sexual orientation had statistically significant effects on self-harm through different mediators. First, age had a statistically significant direct effect on internalized homophobia with a β = –.18, *p* = .002. Internalized homophobia in turn predicted self-harm with a β = .15, *p* = .009. These results thus suggested that being older buffers against internalized homophobia and that consequently this is associated with less self-harm.

Income had a statistically significant direct effect on discrimination with a β = –.23, *p* < .001, which suggested that LGB people of lower income groups reported more discrimination than those in the higher income group. Discrimination in turn had a statistically significant direct effect on self-harm with a β = .15, *p* = .011. Moreover, discrimination impacted on the psychological variable of internalized homophobia with a β = .18, *p* = .002. These results thus suggested that the lower income groups are more vulnerable to discrimination which in turn is associated with more internalized homophobia, culminating in a greater proclivity to self-harm.

It is noteworthy that sexual orientation had a borderline statistically significant impact on LGB victimization with a β = –.11, *p* = .050, suggesting that there is an association between being a sexual orientation minority and LGB victimization. LGB victimization in turn had a significant impact on self-harm with a β = .14, *p* = .016. This result suggested that the more people experience LGB victimization, the more likely they are to self-harm.

Overall, the model supports Hypothesis 5 and suggests that being female, young, of a lower income group and with a lesbian sexual orientation is associated with more discrimination, LGB victimization, internalized homophobia and, ultimately, with self-harm.

## Discussion

Our study focuses on the risk factors for self-harm in an ethnically diverse sample of LGB people in the United Kingdom. At least four socio-demographic groups are at especially high risk of self-harm: those with low income, younger people, females and those who self-identify as lesbians. Although our structural equation model exhibited direct effects of only age and sex on self-harm, with younger and female participants showing greater likelihood of engaging in self-harm, all four variables were indirectly associated with self-harm through discrimination and LGB victimization, which mediated this relationship. This is consistent with the cognitive-behavioral approach to psychopathology (Beck, 1976).

### The risk factors for self-harm

LGB people of lower income were more likely to report discrimination on the basis of their sexual orientation, which in turn put them at increased risk of developing internalized homophobia – itself a correlate of self-harm. It is possible that LGB people of lower income are more reliant on others to survive economically and, thus, are also more susceptible to the (homophobic) attitudes of others. This may be most acute among those from conservative ethnic minority backgrounds in which sexual diversity is less accepted (Jaspal, 2012). Furthermore, being from a lower income background is usually correlated with less educational attainment, which is also a known predictor of conservative attitudes toward sexual orientation (Wright et al., 1999).

Internalized homophobia mediated the relationship between discrimination and self-harm. Discrimination has especially adverse effects on mental health and behavior when it is endorsed and accepted by the LGB individual. Homophobia is likely to be internalized by the individual who is consistently exposed to it, with little or no recourse to alternative representations of their sexual orientation. The LGB individual with lower income will possess less social capital and, thus, find it more difficult to associate with people can who can provide more affirmative representations of their sexual orientation (Barrett & Pollack, 2005), potentially buffering the effect of discrimination on their mental health.

There was a direct impact of age on self-harm. In previous research, it has been suggested that younger LGB people may seek support from others within their peer and age groups, rather than from their parents from whom they may fear negative reactions to their sexual orientation (Jaspal, 2019; Power et al., 2015). In their peer networks, there may be a ‘norm’ in relation to self-harm which motivates others within the group to engage in this behavior as a first-line coping strategy (McManus et al., 2019; Robinson, 2018).

Yet, our model also shows that LGB people of younger age were more likely to manifest internalized homophobia, which in turn increased the risk of self-harm, suggesting that internalized homophobia is a mediating variable. Younger LGB people may be less ‘secure’ in their identities, given that adolescence and early adulthood are characterized by change, adaptation and self-discovery (Mustanski et al., 2013). In the absence of effective social support mechanisms, they may be especially susceptible to internalized homophobia and, thus, self-harm.

Gender had a direct impact on self-harm. This is consistent with previous empirical research indicating that women are generally more vulnerable to self-harm than men (Hawton et al., 2002), with recent research showing that the greatest increase in the incidence of self-harm has been among women (McManus et al., 2019). The reasons for the high prevalence of self-harm in women (compared to men) are not entirely clear – self-harm has been speculatively attributed to the high prevalence of physical abuse from intimate partners, disproportionate exposure to self-identity stressors such as criticism of physical appearance and body mass, and inability to derive social support for coping with stressful situations among women (e.g., Koutek et al., 2016; Stanford et al., 2017).

Sexual orientation had an effect on self-harm through the mediator of LGB victimization. Of all sexual orientation groups, lesbians were the most likely to report LGB victimization, which in turn was associated with self-harm. Authenticity and visibility are important components of identity (Vannini & Franzese, 2008). Yet, it has been found that lesbian women are often less visible than gay men – even in debates about LGBT rights. They may feel that their identities are erased, denied or even ridiculed. In view of this lack of visibility, the impact of LGB victimization may leave them less able to derive social support and, thus, predispose them to construe self-harm as the only viable strategy for channeling their negative emotions in relation to the psychologically stressful experience of victimization.

In addition, our study revealed that self-harm is linked to the presence of depressive symptomatology, such as suicidal ideation and psychological distress (King et al., 2008). Research has consistently shown that females are more vulnerable to depression in comparison to males, which may also explain our finding that self-identified lesbian women are a particularly vulnerable group to both depression and self-harm (see Bebbington, 1996; King et al., 2008).

It must be noted that the focus of the study was not on the specific causes of self-harm among LGB people but rather on the risk factors. The sample was ethnically diverse with varying levels of self-acceptance and experiences of discrimination, victimization and internalized homophobia. In an ethnically diverse sample of 89,199 individuals, it was found that LGB people in every ethnic group represented in the sample were significantly more likely to report engaging in self-harm (Lytle et al., 2014). However, these situational stressors have been found to be more prevalent in BAME communities than in White British communities (Jaspal et al., 2019) and represent high stress-inducing experiences that are associated with self-harm.

### Limitations

This study builds on previous research by providing unique insight into the risk factors for self-harm. There are some limitations which must be acknowledged as researchers take forward this field of study and as practitioners and policymakers engage with these findings.

First, a convenience sample of participants was recruited on online platforms, which precludes empirical generalizability. It is hoped that future research will attempt to recruit more representative participant samples. Second, given the cross-sectional correlational design of the study, the question of causality remains unanswered. Future research should endeavor to shed light on the causes of greater self-harm in women, on one hand, and of greater LGB victimization among lesbian women which in turn is related to self-harm. An experimental design with a manipulation focusing on potential causal variables, such as physical abuse, body image/appearance concerns and inability to derive social support will shed further light on causality. Third, the availability of social support, which is known to buffer the adverse psychological effects of stress-inducing situations, should be examined in future research into self-harm among LGB people. It is plausible to hypothesize that the availability of social support will decrease the incidence of self-harm in those at risk (Jaspal, 2018).

### Clinical implications

Previous research suggests that problem-solving interventions (e.g., Problem Solving Therapy) are highly effective in the treatment of self-harm (Slee et al., 2008). However, given that LGB people are at risk of internalized homophobia, which reflects psychological schemata of self-hatred and self-disgust, it is proposed that problem-solving interventions be bridged with culturally tailored cognitive and behavioral therapies like compassionate-focused therapy (CFT) (Gilbert, 2009) and dialectical behavior therapy (DBT) (see Linehan, 1993; Linehan et al., 2002). Cognitive behavioral therapy alone has had limited effectiveness in LGB populations (Rimes et al., 2019) but, in conjunction with problem-solving interventions, it may be used as a pre-emptive intervention to enhance psychological wellbeing in a population at risk of emotional dysregulation.

Cognitive and behavioral therapies for self-harm regard self-harm as a destructive and dysfunctional coping behavior resulting from inappropriate self-regulation of intolerable affect (e.g., anger and shame), low distress tolerance and impulsivity coupled with the presence of dysfunctional schemata. When imbued with tenets of problem-solving interventions, CFT and DBT may enable the LGB individual to recognize and to challenge dysfunctional cognitions and beliefs, to develop new and adaptive methods of regulating negative affect, and to replace self-harm behavior with more effective problem-solving. In short, individuals may be empowered to transform dysfunctional negative self-cognitions and core beliefs into adaptive and helpful beliefs and self-cognitions.

In view of the centrality of internalized homophobia, the clinical intervention must facilitate feelings of self-compassion and warmth and, thus, the development of a more positive and compassionate view of oneself and others in one’s sexual ingroup (i.e., other LGB people). CFT may be particularly beneficial to LGB people because this group is vulnerable to traumatic experiences of discrimination, which, as our findings demonstrate, can lead to the formation of negative self-schemata (Beck, 1976). Put simply, despite the social progress made in LGB rights, some do live in constant threat and have limited experience of warmth and acceptance in response to their sexual orientation.

The proposed integrative approach would enable the individual to de-activate the threatening emotional regulatory system which can culminate in feelings of anger, fear and depression and associated negative cognitions (e.g., ‘I am worthless’), and promote a self-soothing emotional regulatory system by empowering people to derive compassionate feelings and imagery. By developing self-compassion, LGB people may be empowered to de-activate negative cognitions about themselves and to replace them with feelings and thoughts of self-acceptance, compassion and warmth. This cognitive structure is associated with positive action, rather than self-harm, and is thus potentially conducive to more effective, more sustainable and less-destructive coping strategies in the face of discrimination.
